# Prevalence of diabetes mellitus in newly diagnosed pulmonary tuberculosis in Beira, Mozambique

**DOI:** 10.4314/ahs.v17i3.20

**Published:** 2017-09

**Authors:** Damiano Pizzol, Francesco Di Gennaro, Kajal D Chhaganlal, Claudia Fabrizio, Laura Monno, Giovanni Putoto, Annalisa Saracino

**Affiliations:** 1 Research Section, Doctors with Africa CUAMM; Beira — Mozambique; 2 Clinic of Infectious Diseases, University of Bari, Bari, Italy; 3 Center for Research in Infectious Diseases, Faculty of Health Sciences, Catholic University of Mozambique; Beira - Mozambique; 4 Research Section, Doctors with Africa CUAMM; Padova — Italy

## Abstract

**Introduction:**

Data regarding the association between diabetes mellitus (DM) and tuberculosis (TB) in Africa are scarce. DM screening among TB patients in Mozambique was carried out.

**Methods:**

The study was implemented from January to August 2016 in three Urban Health Centers in Beira, Mozambique and recruited adult (>18 years) patients newly diagnosed with pulmonary TB.

**Results:**

Three hundred and one patients were enrolled (67.4%, males mean age 31.7(SD 11 years). Diabetes was diagnosed in only 3 patients (1%) and impaired glucose tolerance (IGT) in an additional 6 subjects (2%).

**Conclusion:**

A lower than expected prevalence of DM was observed, which could be explained by the lack of traditional risk factors for DM (overweight, age over 45 years, hypertension and smoking) in Mozambique.

## Introduction

Tuberculosis (TB) is one of the leading causes of death worldwide. Mozambique represents one of the most affected countries in the world, with an estimated prevalence of over 500 per 100,000 population^1^. Although great efforts have already been made, the way to defeat TB remains long. The WHO End TB Strategy aims to a 90% reduction in TB deaths and an 80% reduction in the TB incidence rate by 2030^1^.

Many factors contribute to the lack of efficacy in containing TB, as social determinants of health (SDH) and concomitance of other co-morbidities, such as co-infections (in particular with HIV) and non-communicable diseases, such as diabetes mellitus (DM)^2^.

SDH, which can be defined as conditions in which people are born, grow, live, work and get old, are strongly influenced by the distribution of wealth, power, resources and they have an immediate impact on health^2^. In fact, it is well known the association between low socio-economic levels and TB^3^. Furthermore, TB is the most common opportunistic infection among HIV-positive patients and, especially in sub-Saharan Africa, the burden of HIV/TB co-infection is the highest worldwide, with almost 80% of all cases of incident TB in persons with HIV infection^4^.

Growing evidence suggests that TB and DM represent a mutual risk factor of occurrence of each other^5^, causing also a reciprocal worsening, due to both pathogenic mechanisms^6^, and metabolic factors^7^. In fact, if on one hand patients with DM have a higher risk of acquiring TB, on the other hand, patients with DM and TB have a high likelihood of delayed diagnosis, healing, and increased severity of symptoms and mortality for both diseases^8^. Nowadays, sub-Saharan Africa and low income countries are increasingly affected by DM^9^–^10^ and, the increasing frequency of DM co-existing with TB and HIV, is an example of interaction between communicable and non-communicable diseases, requiring a multidisciplinary and integrated approach^8^. In Mozambique, data on the prevalence of DM in TB patients are limited. The few data available on DM in this country in the general population shows a national prevalence of 2.9%^11^–^12^, and people are often unaware of their condition^11^–^12^. The present study aimed to assess the prevalence of DM in patients newly diagnosed with pulmonary TB in Beira, the second largest city in Mozambique, to better define the relationship between TB and DM.

## Patients and methods

### Study design and population

This study took place in three urban health centres of Beira city involved in the National Tuberculosis Control Programme (PNCT): Ponta-Gea, Munhava and Macurungo, that are largest in Sofala's Province. The study was implemented from January to August 2016 and we recruited a total of 301 patients who were newly diagnosed with pulmonary TB. Inclusion criteria were: age ≥18 years, a confirmed TB diagnosis (positive sputum smear result or GeneXpert positive result or culture positive result) and starting of anti-TB treatment. Exclusion criteria were: previous TB treatment or TB treatment started within the previous two months. Informed consent was requested from all enrolled patients. Anti-TB treatment was prescribed according to guidelines of the National Tuberculosis and Leprosy Control Programme of Mozambique^13^.

### Methods

A face-to-face interview conducted by a trained nurse encompassed questions about demographic characteristics (age, residence, education, occupation, marital status, monthly income), possible pregnancy, risk behaviours (sexual behaviour/partnerships, concurrent sex partners, condom use, smoke and alcohol abuse, etc.) and medical history, including TB and diabetes symptoms. A basic physical examination (vital signs, weight, height, waist circumference, blood pressure and general appearance) was performed. The body mass index (BMI) was calculated. Moreover, subjects with unknown HIV status received a pre-HIV test, other suggestion to undergo a rapid HIV test, if not done before. For the diabetes diagnosis, two consecutive fasting blood glucose tests were performed to each participant. We did a screening and diagnosis for TB infection according to clinical, microbiological and radiological algorithm established by the WHO Guidelines for patients with or without HIV infection^1^ and in patients with TB infection, the clinical severity was evaluated based on extension, site and number of localizations of disease (pulmonary/extrapulmonary). According to the WHO guidelines, patients were considered as non-diabetic if both measurements were ≤110 mg/dl, and as diabetic if both measurement were above 126 mg/dl. If at least one value was between 110 and 126 mg/dl, the Oral Glucose Tolerance Test (OGTT) was performed: patients were considered diabetic when plasma glucose at 2 hours was ≥200 mg/dl^14^.

All TB-diabetic patients underwent fundus examination, urinary stick and assessment of diabetic complications.

### Statistical analysis

Data obtained from the answers to the questionnaire were imported on Excel 5.0 and analysed using the STATA 13.0 software.

Mean and standard deviation for continuous variables and frequency for categorical variables were calculated as descriptive statistics. Due to the small number of diabetic patients, a comparison between diabetic and non-diabetic subjects was not possible.

### Ethical approval

The study was approved by the Comité Nacional de Bioetica para a Saúde/ National Bioethics Committee for Health by the protocol Ref: 168/CNBS/15.

## Results

### Socio-demographic characteristics

The social-demographic characteristics of the study population and patients' life style are reported in Table 1. The 301 patients with a new diagnosis of pulmonary TB included 203 males (67.4%) and 98 females (32.6%), mean age 36.7 years. Nearly a half 141 patients (47.5%) had no educational degree; 157 (52.9%) patients were employed and only 10 (3.8%) had a monthly income higher than 100 Euros. Only 7 patients (2.4%) claimed to have more than one sexual partner and 32 (11.3%) stated to always use condoms. The majority of subjects 274 (93.2%) declared non smoking while alcohol consumption was moderate, and only 2 patients (0.7%) were daily drinkers.

**Table 1:** Socio-demographic and lifestyle characteristics

Variable	N. (%)
**Number of cases examined**	301
**Gender**	
**Female**	98 (32.6%)
**Male**	203 (67.4%)
**Age**	36.7
**Mean age (years)**	
**Range (years)**	18 – 83
**Educational degree**	
**None**	141 (47.5%)
**Primary school**	125 (42.1%)
**Secondary school**	25 (8.4%)
**University degree**	6 (2.0%)
**Occupation**	
**Unemployed**	95 (32%)
**Housewife**	18 (6%)
**Student**	27 (9.1%)
**Worker**	157 (52.9%)
**Monthly income (Euro)**	
**None**	106 (40.8%)
**< 25**	29 (11.2%)
**25–50**	53 (20.4%)
**50 – 100**	62 (23.8%)
**> 100**	10 (3.8%)
**BMI**	
**Severe thinness**	59 (19.6%)
**Moderate thinness**	32 (10.6%)
**Light Thinness**	78 (26%)
**Healthty**	128 (42.5%)
**Overweight**	4 (1.3%)
**Sexual partner/s**	
**No**	110 (37.4%)
**One**	177 (60.2%)
**More than one**	7 (2.4%)
**Condom Use**	
**Never**	131 (46.1%)
**Sometimes**	121 (42.6%)
**Always**	32 (11.3%)
**Smoking**	
**No**	274 (93.2%)
**1–5/day**	15 (5.1%)
**20/day**	5 (1.7%)
**Alcohol drinking**	
**No**	260 (87.8%)
**Once a week**	34 (11.5%)
**Every day**	2 (0.7%)

### Clinical and laboratory characteristics

The main laboratory and clinical characteristics are shown in Table 2. The most sensitive test for TB diagnosis was GeneXpert, which was positive in 56 of 58 (96.6%). A chest X-ray was performed for 155/301 subjects and 148 of 155 (95.5%) showed TB-compatible lesions.

**Table 2:** Clinical and laboratorial characteristics

Variable	N. (%)
**Sputum Examination**	
**Negative**	50 (16.9%)
**Positive**	246 (83.1%)
**Sputum colture**	
**Negative**	3 (75%)
**Positive**	1 (25%)
**GeneXpert**	
**Negative**	2 (3.4%)
**Positive**	56 (96.6%)
**Chest X-ray**	
**Negative**	7 (4.5%)
**Positive**	148 (95.5%)
**HIV**	
**Negative**	121 (42.9%)
**Positive**	161 (57.1%)
**ART**	
**Yes**	66 (26.7%)
**No**	181 (73.3%)
**Diabetes**	
**Yes**	3 (1%)
**No**	290 (97%)
**IGT**	6 (2%)
**High Blood Pressure**	
**Yes**	1 (0.3%)
**No**	293 (99.7%)

The HIV status was known for 282 patients: of them, 161 (57.1%) were positive. Sixty-six patients (26.7%) were on anti-retroviral treatment (ART). The BMI was below healthy range in 169 (55.6%) whereas 4 subjects (1.3%) were overweight. One individual (0.3%) had hypertension.

Diabetes was diagnosed in only 3 patients (1%) and IGT in additional 6 subjects (2%). Nobody was aware of their diabetic status.

Reported clinical signs and symptoms are summarized in Table 3. The most frequent TB symptoms were: cough (90.9%), weight loss (88.2%), asthenia (58.8%) and fever (58.8%). The patients with DM and IGT reported weight loss, polyuria and asthenia.

**Table 3:** Main symptmoms reported from patients with pulmonary TB

Symptoms	N. (%)
**No symptoms**	3 (1%)
**Cough**	269 (90.9%)
**Fever**	174 (58.8%)
**Astenia**	215 (72.6%)
**Dyspnea**	95 (32.1%)
**Hemoptysis**	4 (1.4%)
**Night sweats**	118 (39.9%)
**Weight loss**	261 (88.2%)
**Polyuria**	13 (4.4%)
**Polydipsia**	7 (2.4%)
**Polyphagia**	11 (3.7%)

## Discussion

In sub-Saharan Africa, there is an emerging strong correlation between communicable and non-communicable diseases due to the long lasting presence of epidemic infections such as HIV and TB and the increasing incidence of chronic diseases as DM and hypertension. In 2030, people living with diabetes in Africa are estimated to rise from 21.1 million in 2010 to up to 23.9 million^15^. In the same area, DM causes about 4.9 million deaths per year and approximately 76% of these deaths occur in people aged less than 60 years^9^. Diabetes is considered among priorities in the National Strategic Plan for the prevention and control of non communicable diseases also in Mozambique^13^. However, the prevalence of DM seems to be quite low, around 3% in the general population aged from 25 to 64 years old, and it is higher among people living in rural areas^11^. Our study showed a low prevalence of DM in newly TB-diagnosed patients (1%) compared to data regarding the general population, although a disorder in glycometabolic control was found in 3% of subjects.

This finding in our population could be explained by the lack of some of the risk factors recognised for the onset of DM, including overweight, age over 45 years, hypertension and smoking^14^. In fact, in our population the mean age was 36.7 with only few patients overweight and affected by hypertension. Indeed, two out of three diabetic patients in our study were older than 45 years and were smokers, whereby one subject was HIV positive. Among the six patients with IGT the median age was 32, there was no smoking, three were HIV positive and one consumed alcohol.

In low-income countries, the association between TB and DM is variable, ranging from 1.9% to 35%^14^. Other authors reported a higher incidence of DM in TB patients from African countries or low income settings^10^–^16^. On the contrary, other studies showed a low association of DM and TB in low-income settings^17^, which was explained by the young age of enrolled patients, similarly to the results of the present study^18^–^19^.

Furthermore, this study underlines the importance of other factors associated with the onset of TB, including SDH. The phenotype of newly diagnosed patients with pulmonary TB in Beira was outlined: patients are mainly young, underweight, with a low educational background and low income, without smoke nor alcohol habits, basically without hypertension and diabetes mellitus but in most cases living with HIV infection. According to our data, SDH appear to influence the onset of TB but also the frequency of MDR and the adherence to therapy^3^. HIV is the most well known disease associated with TB; and our data confirms this correlation as almost 60% of patients with a known HIV status were seropositive. On the other hand, additional risk factors for TB such as smoking and alcohol use, are almost inexistent in our study. A limitation of our study is that, our data could be biased because they were not verified and were based on personal interview. Another drawback of the study is the small sample size and lack of a control group.

## Conclusion

Our study shows a low prevalence of DM in newly diagnosed patients with pulmonary TB in Mozambique. The lower than expected DM prevalence, however, does not clash with the hypothesis of a mutual interaction existing between these two diseases. Future studies enrolling a greater number of diabetic patients would be crucial in order to assess definitive epidemiological data to contribute to the TB End Strategy in one of the most affected countries like Mozambique.
